# Prescription psychostimulants or atomoxetine and the risk of stimulant-related hospital admissions in adults with and without stimulant use disorder: a Swedish population-based within-individual observational study

**DOI:** 10.1016/j.lanepe.2026.101658

**Published:** 2026-03-24

**Authors:** Patrick Bach, Johan Franck, Jonas Hällgren, Härje Widing, Mika Gissler, Jeanette Westman

**Affiliations:** a Department of Clinical Neuroscience, Karolinska Institutet, Stockholm, Sweden; b Department of Addictive Behavior and Addiction Medicine, Central Institute of Mental Health, University of Heidelberg, Medical Faculty Mannheim, Germany; c Academic Primary Care Centre, Region Stockholm, Sweden; d Department of Neurobiology, Care Sciences and Society, Karolinska Institutet, Stockholm, Sweden; e Department of Data and Analytics, Finnish Institute for Health and Welfare, Helsinki, Finland; f Department of Molecular Medicine and Surgery, Karolinska Institutet, Stockholm, Sweden; g Department of Health Care Sciences, Marie Cederschiöld University, Stockholm, Sweden

**Keywords:** Psychostimulants, Stimulant use disorder, Stimulants, Hospitalization, Pharmacovigilance, Register-based study

## Abstract

**Background:**

Concerns exist that psychostimulant treatment increases the risk of misuse, particularly in individuals with a history of stimulant use disorder (SUD). This study assessed changes in the risk of stimulant-related hospitalizations after initiating treatment with psychostimulants or atomoxetine in individuals with and without SUD.

**Methods:**

This population-based study used Swedish registers to identify adults with and without history of SUD, who used psychostimulants or atomoxetine, a non-stimulant prescription medication, between 2008 and 2021. A within-individual design was used to compare the rate ratios (RR) of stimulant-related hospitalizations in two six months periods before and after treatment initiation. Stimulant-related hospitalizations were defined as any hospital visit with a stimulant-related main diagnosis.

**Findings:**

Of the 132,666 adults receiving psychostimulants or atomoxetine, 2·4% (3161/132,666) had a history of SUD, while 97·6% (129,505/132,666) did not. The RR for stimulant-related hospitalizations in the six months following treatment initiation was 0·79 (95% CI 0·62–0·99) for individuals with a history of SUD and 0·84 (0·70–1·02) for those without, compared to the 6-month period before treatment initiation. Results were similar across sexes, age groups, and medication classes and sensitivity analyses comparing 8-/12-/52-week periods before and after treatment initiation were consistent with the main findings.

**Interpretation:**

We did not observe higher rates of stimulant-related hospitalizations in the 6-month period after initiation of treatment with psychostimulants or atomoxetine in individuals with or without a history of SUD. The observed associations are consistent with a reassuring safety profile and may help inform clinical decision-making when considered alongside clinical guidelines.

**Funding:**

This research was funded by 10.13039/501100006636FORTE (2020-00467), 10.13039/501100004047Karolinska Institutet (KID, 2020-02653), and Region Stockholm (FoUI-977024).

**Research in context**

**Evidence before this study**

Prior research raised concerns that psychostimulants may increase the risk of misuse and hospitalizations, especially in individuals with stimulant use disorder. On the other hand, clinical studies have provided evidence that treatment with psychostimulants and the non-stimulant drug atomoxetine can reduce the use of illicit stimulants and subsequent hospitalization risk. We searched PubMed for studies published in English between 1 January 2000 and 20 February 2025 using combinations of the search terms “psychostimulants, stimulants, (dex-)methylphenidate, atomoxetine, (lis-)dexamphetamie or (ar-)modafinil” and “misuse, abuse, intoxication or hospitalization”. We also reviewed clinical guidelines from national bodies, including U.S. and European Practice Guidelines for the treatment of attention deficit hyperactivity disorder (ADHD) and comorbid stimulant use disorder. There is no clear consensus about whether psychostimulant treatment, particularly in individuals with a history of stimulant use disorder, increases the risk of abuse, misuse, and subsequent hospitalizations. Even though studies indicated that psychostimulant treatment might not increase substance abuse risk per se, the risk of misuse and intoxication is a concern in individuals, which present with a history of a stimulant use disorder (SUD).

Clinical guidelines reflect this uncertainty. UK NICE guidelines recommend careful monitoring, but do not contraindicate stimulants in SUD, while German guidelines consider history of SUD as a potential contraindication. Spanish clinical practice guidelines provide weak recommendations for psychostimulants in ADHD with comorbid SUD, emphasizing individualized assessment. At the same time, several clinical studies suggest that treatment with dexamphetamine, or methylphenidate or atomoxetine could reduce the use of illicit stimulants in those with SUD and observational studies also reported associations between stimulant treatment, specifically lis-dexamphetamine, and lower risk of substance use-related mortality in individuals with SUD. Still, the reported treatment effects were variable and not consistent across studies. Studies varied substantially in design, population characteristics, follow-up duration, and outcome definitions, limiting definitive conclusions about real-world safety profiles, so a comprehensive analysis of large datasets has the potential to contribute important information on the risk of stimulant-related hospitalizations after initiation of psychostimulant or atomoxetine treatment.

**Added value of this study**

We examined the risk of stimulant-related hospitalizations in 132,666 individuals after initiation of psychostimulant or atomoxetine treatment using a within-individual comparison design that controlled for time-invariant confounding. Results evidence lower rates of stimulant-related hospitalizations after initiation of treatment with psychostimulants or atomoxetine in individuals with or without a history of SUD compared to before. Notably, results were similar across sexes, age groups, and medication groups.

**Implications of all the available evidence**

Our findings refute concerns that psychostimulant treatment should be avoided in individuals with a history of stimulant use disorder. It is important to confirm these findings in controlled clinical studies that can define treatment periods, doses, and co-treatments more precisely and that control for changes in a person's characteristics over time, such as psychosocial factors and comorbidities.

## Introduction

The use of prescription psychostimulants, such as methylphenidate and dexamphetamine, is increasing over the last years in Sweden^1^ and the risk of overuse and misuse of such medication and resulting physical and mental harm is an increasing public health concern. Data of the Swedish Prescribed Drug Register show that between 2006 and 2023, the prevalence of prescription stimulant use rose from 0·83 to 23·7 per 1000 inhabitants aged 20 through 64 years. The substantial increase in the prescription of stimulants observed in Sweden is consistent with global trends. International studies across 64 countries found that stimulant prescriptions increased by about 9·7% annually between 2015 and 2019, with the highest rates observed in North America, Oceania, and Northern/Western Europe, with recent data from England evidencing that prescriptions for stimulants increased by 18% annually from 2019 to 2020 to 2023–2024, rising from 25·2 to 41·6 per 1000 individuals.^1^, ^2^, ^3^, ^4^, ^5^ These parallel trends across diverse healthcare systems suggest that increased societal awareness, evolving diagnostic practices and treatments of attention deficit hyperactivity disorder (ADHD) might contribute to the global increases in ADHD medication use. Further, a study of Swedish national data indicated that about 7·6% of individuals who received psychostimulants used about 1·5 times the recommended maximum daily dose, and the odds of overuse were even higher in those who had used psychoactive substances in the past.^6^ Studies on U.S. *data* reported misuse in about 25% of individuals receiving prescription stimulants and found that the risk for stimulant abuse was associated with a history of nonmedical use of stimulants.^7^ Further studies reported that a history of substance abuse, access to prescription stimulants and misuse of prescription psychostimulants are significant risk factors for psychostimulant abuse, especially in males and users of amphetamine-like stimulants.^8^ And even though studies indicated that psychostimulant treatment might not increase substance abuse risk per se,^9^ the risk of misuse and intoxication is a concern in individuals, which present with a history of a stimulant use disorder (SUD).^10^

Given these findings, concerns have been raised that psychostimulants might increase the risk of stimulant-related hospitalizations. Treatment guidelines for ADHD recommend that all patients should be assessed for symptoms of substance abuse,^11^^,^^12^ and clinicians should carefully monitor signs of misuse.^11^^,^^12^ Some guidelines even consider the use of stimulants, especially amphetamine-like agents, to be contraindicated in patients with a history of SUD.^11^ As a result, patients with a medical indication for psychostimulant treatment and who would benefit from it might not receive it. Evidence from clinical trials and observational studies has made this challenge of managing the potential benefits and risks of psychostimulant prescription even more pressing, especially in patients with a history of SUD. Further studies suggest that especially high doses of psychostimulants, reduce the use of illicit stimulants in those with SUD,^13^, ^14^, ^15^, ^16^, ^17^, ^18^, ^19^ while other studies questioned a positive risk-benefit-ratio of high unlicensed doses.^20^ Specifically, prescription stimulants like dexamphetamine^13^^,^^14^^,^^19^ and methylphenidate^15^, ^16^, ^17^, ^18^, ^19^ have had positive but variable effects in reducing stimulant use and injury risk, whereas atomoxetine has yielded less consistent results.^21^ Additionally, Swedish nationwide cohort studies reported associations between lis-dexamphetamine treatment and lower risk of hospitalizations, lower substance use-related and all-cause mortality in a cohort of more than 13,000 individuals with SUD^22^ and between treatment with ADHD medication and lower mortality in a cohort of 148,578 individuals with ADHD^23^ and lower risks of self-harm, injury, traffic crashes and crime in a cohort of 247,420 ADHD medication users.^24^ This creates profound therapeutic uncertainty for patients with both ADHD and SUD history. While they may be at theoretical risk for stimulant-related complications if prescribed psychostimulants for ADHD, they also might benefit from such treatment, potentially including reduced substance use.

The primary aim of our study was thus to examine the risk of stimulant use-related hospitalizations after initiation of treatment with psychostimulants or atomoxetine in individuals with and without a history of a SUD. The secondary aim was to investigate risk across sexes, age groups, and different medication classes.

## Methods

### Study design and sample

**Fig. 1:**
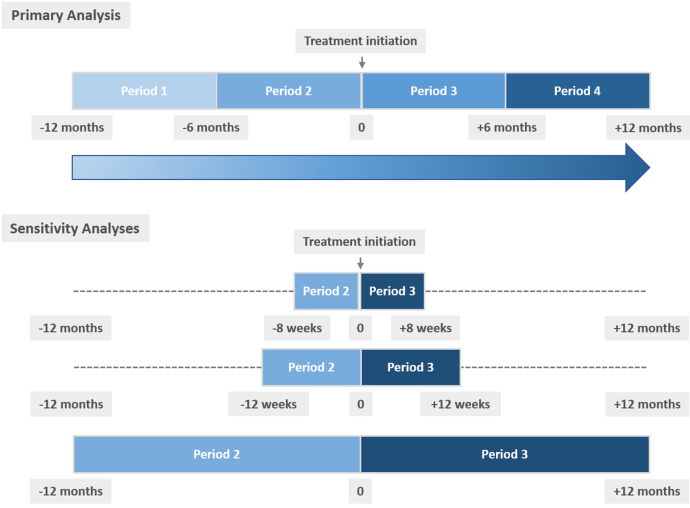
Illustration of the study periods relative to treatment initiation in the primary and sensitivity analysis.

This population-based cohort study applied a within-individual study design to compare the rate of hospitalizations due to stimulant use in adults with and without history of SUD before and after initiation of treatment with prescription of either psychostimulants (e.g., methylphenidate, dexamphetamine) or atomoxetine, a non-stimulant selective norepinephrine reuptake inhibitor. Although pharmacologically distinct, both classes are first-line ADHD medications and are considered together in clinical decision-making regarding treatment safety. History of SUD was defined as having at least one registered inpatient or outpatient visit with a diagnosis of stimulant use disorder (ICD-10 codes F14–F15) during the 5-year period from seven to two years before treatment initiation. To this end, we compared the rate of hospitalizations during four six-month observation periods (P): P1 (month −12 to month −6 before treatment initiation), P2 (month −6 to the day before treatment initiation), P3 (day of treatment initiation to the day before month +6 after treatment initiation), and P4 (month +6 to month +12 after treatment initiation) (see Fig. 1). In line with methodological guidance for self-controlled case series studies, risk period length was chosen based on clinical pharmacology and prior evidence. Six-month intervals were selected to encompass titration, symptom stabilization and early maintenance, consistent with guideline recommendations monitoring of ADHD medication.^11^^,^^12^

Using the Swedish national registers, we identified adults who received a new prescription for psychostimulants or atomoxetine between 2008 and 2021 and were aged 18 through 64 years. New users were defined as individuals who, according to the Swedish Prescribed Drug Register, were first dispensed psychostimulants or atomoxetine between 1 January 2008 and 31 December 2021, considering prescriptions for methylphenidate (Anatomical Therapeutic Chemical [ATC] code N06BA04), dexamphetamine (N06BA02), metamphetamine (N06BA03), modafinil (N06BA07), atomoxetine (N06BA09), dexmethylphenidate (N06BA11), lisdexamphetamine (N06BA12), or armodafinil (N06BA13). New users were defined as individuals who initiated treatment with psychostimulants during the observational period and who had not received any of these medications for at least 2·0 years prior to the first registered dispensation. Individuals were excluded if they had immigrated to Sweden after the start of P1 or died or emigrated before the end of P4.

### Data sources

The data for this cohort study were collected from registers with nationwide coverage. The Swedish National Prescribed Drug Register, which contains data on all drugs that have been dispensed in Sweden since 1 July 2005, was used to obtain data on dispensation dates, ATC codes (see above), and defined daily doses (DDDs). The Total Population Register was used to obtain information on sex, age, and migration. The National Patient Register provided information on inpatient visits and specialized outpatient services and the associated main diagnoses, which are coded according to the tenth revision of the International Statistical Classification of Diseases and Related Health Problems (ICD-10). The National Cause of Death Register was used to identify and exclude individuals who died during the observation period.

### Outcomes

Stimulant use-related hospitalization was the primary outcome. It was defined as hospital admission with a main diagnosis of a stimulant-related disorder (ICD-10 codes F14·x or F15·x, Appendix p2), including acute intoxication, harmful use, dependence syndrome, withdrawal state with and without delirium, stimulant-related psychotic disorders, stimulant-related amnesic syndromes, stimulant-related residual and late-onset psychotic disorders, and other and unspecified mental and behavioral disorders due to stimulant use.

As a secondary outcome, we used hospitalizations due to stimulant-related acute intoxication, harmful use, dependence syndrome, and withdrawal states with and without delirium (ICD-10 codes: F14·0-F14·4 or F15·0-F15·4), thus excluding psychotic syndromes, amnestic syndromes, and other and unspecified syndromes.

When the registered hospitalization periods overlapped or discharge and admission were registered on the same day (e.g., because of transfer from one to another ward), the event was not considered a new hospitalization.

### Statistical analysis

To investigate the risk of stimulant-related hospitalizations after initiation of treatment, our primary analysis compared the rate of these events during the two 6-month periods on either side of treatment initiation (see Fig. 1), referred to as period 2 (P2) and period 3 (P3). Our secondary analyses also compared the rate of stimulant use-related hospitalizations during the 6-month period immediately before psychostimulant initiation (P2) with that during the 6-month period starting at month 6 after treatment initiation and running until month 12 after treatment initiation (P4, see Fig. 1). For completeness, we also compared the rate of stimulant use-related hospitalizations during the 6-month period after treatment initiation (P3) with that during the 6-month period that started one calendar year before treatment initiation (P1).

Following previous studies,^25^ a conditional Poisson regression model was used to estimate the rate ratios (RR) of stimulant-related hospitalizations during the different 6-month study periods with each patient as a separate stratum. Only individuals who had at least one stimulant-related hospitalization during either of the observation periods contributed data to the analysis. We performed the primary (P3 versus P2) and secondary analyses (P3 versus P4 and P3 versus P1) separately in patients with and without a history of stimulant use disorder. The results are presented as rate ratios (RRs) with 95% confidence intervals (CIs). Hospitalization rates were calculated as the number of stimulant-related hospitalizations per 10,000 person-weeks.

The influence of individual characteristics that do not change over time (e.g., sex, baseline disease severity) was adjusted for by conducting within-individual comparisons. To test the robustness of results from comparing 6-month periods before and after treatment initiation, we performed sensitivity analyses in which we set the length of the observation period to eight, twelve weeks and twelve months before and after treatment initiation instead of six months (Fig. 1).

As sensitivity analyses, we also investigated the rate of stimulant-related hospitalizations before and after initiation of treatment stratified by sex (males, females), stratified by age group (18–34, 35–49, 50–64 years), stratified by medication group, and stratified by the amount of prescribed defined daily doses (DDDs) during the year after treatment initiation.

Data management and statistical analyses were done with SAS version 9·4 and R software version 4·4·3 (R Foundation for Statistical Computing, Vienna, Austria).

### Ethics approval

The study was approved by the Regional Ethical Review Board in Stockholm (Stockholm, Sweden, decision id 2019-00516). No informed consent was required since no registered person was contacted, and only anonymized data were used in the study. Thus, individual records were unidentifiable during the analysis.

### Role of the funding source

The funder of the study had no role in study design; data collection, analysis, or interpretation; or in writing the report.

## Results

We identified 132,666 individuals who had started treatment with psychostimulants between 2008 and 2021 and met the study inclusion criteria. The cohort included 3161 (2·4% [3161/132,666]) individuals with a history of stimulant use disorder (SUD) and 129,505 (97·6% [129,505/132,666]) without such a history (Table 1). Of those with a history of SUD, a total of 304 (9·6% [304/3161]) had at least one stimulant-related hospitalization during the 2-year observational period, whereas 647 (0·5% [647/132,666]) in the group without a history of SUD had at least one SUD-related hospitalization. Differences between individuals with and without history of SUD on sociodemographic characteristics, number of stimulant-related hospitalizations, prescriptions of different medication classes, dispensed DDDs, number of dispensing events in the year after treatment initiation, comorbid substance use disorders, and co-dispensing of other psychoactive medications ±3 days before and after treatment initiation are presented in Table 1.

**Table 1:** Characteristics of the study population (N = 132,666), which included all Swedish residents aged 18 through 64 years who received a prescription for psychostimulants or atomoxetine between 2008 and 2021, depicted separately for individuals with and without history of a stimulant use disorder.

	With a history of SUD n = 3161	Without a history of SUD n = 129,505	p
Age, mean (SD)	36·28 (10·0)	33·97 (10·9)	<0·001
Sex, n (%)			<0·001
Male	2088 (66·1)	64,788 (50)	
Female	1073 (33·9)	64,717 (50)	
Psychostimulant class prescribed at treatment initiation, n (%)			<0·001
Dexamfetamine, lisdexamfetamine, or metamfetamine	383 (12·1)	12,852 (9·9)	
Atomoxetine	583 (18·4)	14,322 (11·1)	
Methylphenidate or dexmethylphenidate	2151 (68)	91,043 (70·3)	
Modafinil or armodafinil	44 (1·4)	11,288 (8·7)	
DDDs first year, mean (SD)	694·01 (709·7)	445·08 (431·1)	<0·001
Number of dispensing events in the first year after treatment initiation, mean (SD)	11·30 (8·9)	8·75 (6·3)	<0·001
Number of hospital admissions during the 2-year study period, n (%)			<0·001
0	2857 (90·4)	128,858 (99·5)	
1–2	244 (7·7)	608 (0·5)	
3–5	50 (1·6)	32 (0)	
≥6	10 (0·3)	7 (0)	
Main diagnosis for hospital admissions, n (%)			<0·001
Intoxication/abuse/addiction/withdrawal syndrome (ICD-10 codes: F14 or F15·0, F15·1, F15·2 F15·3, F15·4) Acute intoxication, harmful use, dependence syndrome, withdrawal state with and without delirium due to stimulant use (ICD-10 codes: F14 or F15·0, F15·1, F15·2 F15·3, F15·4)	411 (71)	596 (70·7)	
Stimulant-related psychotic disorders, and late-onset or residual psychotic disorders (ICD-10 codes: F14 or F15·5, F15·7)	131 (22·6)	207 (24·6)	
Stimulant-related amnesic syndromes, and other and unspecified mental and behavioral disorders due to stimulant use (ICD-10 codes: F14 or F15·6, F15·8, F15·9)	37 (6·4)	40 (4·7)	
Psychopharmaceutical classes dispensed within ± 3 days of initiation of treatment with psychostimulants, n (%)			
N02A	85 (2·7)	2411 (1·9)	<0·001
N02B	189 (6)	4151 (3·2)	<0·001
N03A	135 (4·3)	3048 (2·4)	<0·001
N05A	356 (11·3)	4597 (3·5)	<0·001
N05B	255 (8·1)	6965 (5·4)	<0·001
N05C	520 (16·5)	14,746 (11·4)	<0·001
N06A	391 (12·4)	13,933 (10·8)	0·0039
N07B	159 (5)	1090 (0·8)	<0·001

Legend: ICD-10 = International Statistical Classification of Diseases and Related Health Problems 10th Revision, SUD = stimulant use disorder.

**Fig. 2:**
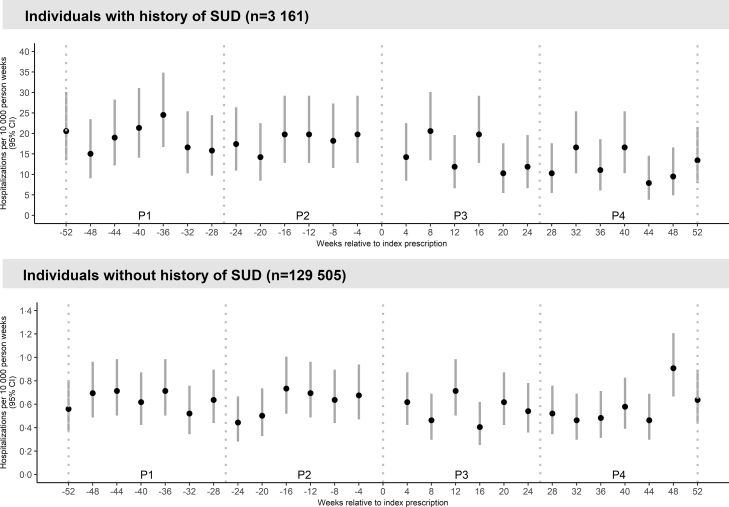
Rate of stimulant use-related hospital visits, defined as a main diagnosis of F14.x or F15.x according to the ICD-10, per 10,000 person-weeks during the 52 weeks before and after psychostimulant treatment initiation in individuals with a history of a stimulant use disorder (n = 3161) and individuals without a history of a stimulant use disorder (n = 129,505) (CI = confidence interval, p = period).

The overall rate of stimulant-related hospitalizations was 17·76 events per 10,000 person-weeks (95% CI 16·16–19·05) in those with a history of SUD, and 0·62 events per 10,000 person-weeks (0·58–0·67) in those without a history of SUD. The rates of hospitalizations attributed to stimulant use in the 52 weeks before and after initiation of psychostimulant treatment for those with and without a history of SUD are shown in Fig. 2.

**Fig. 3:**
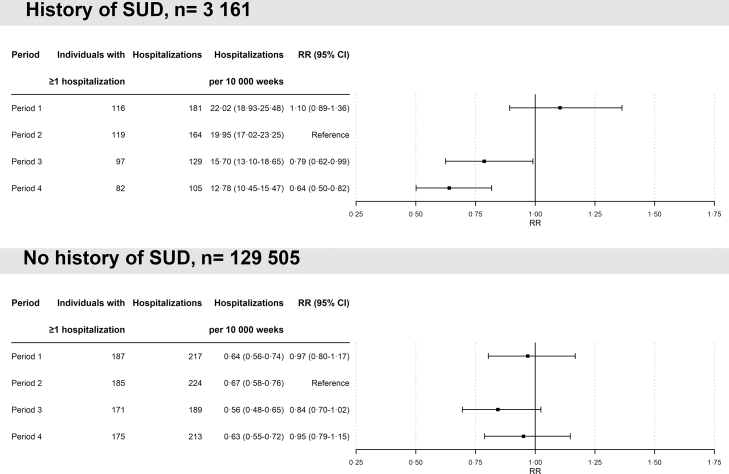
Relative risk of stimulant use-related hospitalizations in individuals with (n = 3161) and without (n = 129,505) history of a stimulant use disorder during different periods before (period 1) and after initiation (periods 3 and 4) of psychostimulant treatment (reference: period 2). RR = rate ratios.

The primary comparisons of the 6-month period right before treatment initiation (P2) and the 6-month period after treatment initiation (P3) showed a lower risk of stimulant use-related hospitalizations after treatment initiation among individuals with a history of SUD (P3 versus P2: RR 0·79, 95% CI 0·62–0·99, Fig. 3). Among those without a history of SUD, we found a non-significant reduction in the risk of stimulant use-related hospitalizations from before to after treatment initiation (P3 versus P2: RR 0·84, 95% CI 0·70–1·02, Fig. 3).

For the secondary comparison of P2 with P4, we found a significantly lower risk of stimulant use-related hospitalizations after treatment initiation among individuals with a history of SUD (P2 versus P4: RR 0·64, 95% CI 0·50–0·82, Fig. 3), and a non-significant lower risk among individuals without SUD (P2 versus P4: RR 0·95, 95% CI 0·79–1·15, Fig. 3).

Additional analyses that set the length of the observation periods P2 and P3 before and after treatment initiation to either eight weeks, twelve weeks or twelve months showed a lower hospitalization risk after initiation of treatment among the group with a history of SUD and among the group without a history of SUD for all period lengths (Appendix pp3–5). However, only the comparison of the twelve-month periods before and after treatment initiation among individuals with history of SUD yielded significance (P2 versus P3: RR 0·68, 95% CI 0·57–0·80).

Stratification by medication class indicated lower hospitalization risks after initiating (dex-)methylphenidate in individuals with prior SUD, and when considering the psychostimulants (dex-)methylphenidate and (lis-)dexamphetamine as combined group (Appendix p8). Other comparisons showed no significant change in hospitalization risk. Among individuals without SUD, hospitalization risk was lower after initiation of atomoxetine treatment, while other comparisons showed no significant change in hospitalization risk (Appendix p9). Because there were few events (<9 per period), results for modafinil/armodafinil could not be estimated meaningfully and are hence not presented.

Stratification of analyses by sex corroborated the findings of the primary analyses and indicated a lower risk of stimulant use-related hospitalizations after treatment initiation in males and females with prior SUD (Appendix p10). No significant change in risks were found in males and females without history of SUD (Appendix p11).

Stratification of analyses by age groups indicated a significantly lower risk of stimulant-related hospitalizations after treatment initiation among individuals with prior SUD in the age groups 50–64 and a descriptively lower risk in individuals aged 35–49, but not in individuals aged 18–34 (Appendix p12). Among individuals without prior SUD, we observed no significant change in risk, but point estimates indicated a descriptively lower risk after treatment initiation in the age groups 35–49 and 50–64 (Appendix p13).

In addition, we conducted an additional sensitivity analysis in individuals with history of SUD, adjusting for time since the initial stimulant use disorder diagnosis (in 6-month intervals, i.e. 0–6, 6–12, etc.), to capture potential disease maturation effects. Adjusting for time since diagnosis did not alter our findings (Appendix p7).

## Discussion

This study examined the risk of stimulant-related hospitalizations in 132,666 individuals. In agreement with previous observational studies among individuals with amphetamine use disorder^22^ and ADHD^26^ and clinical trials investigating effects of dexamphetamine^13^^,^^14^^,^^19^ and methylphenidate in SUD,^15^, ^16^, ^17^, ^18^, ^19^ we did not observe a higher rate of stimulant-related hospitalizations after initiation of psychostimulant treatment, neither in individuals with a history of SUD, nor in individuals without history of SUD. In contrast, during the first 6-month period after treatment initiation, we observed a 20% lower risk of stimulant-related hospitalizations among individuals with a history of SUD. Our findings challenge concerns that psychostimulant treatment increases the risk of stimulant-related hospitalizations, particularly in individuals with a history of SUD. Instead, we observed a lower hospitalization rate following treatment initiation—primarily among those receiving (dex-)methylphenidate—consistent with clinical trials showing reduced stimulant use in treated individuals with SUD.^13^, ^14^, ^15^, ^16^, ^17^, ^18^, ^19^ Exploratory analyses further indicated that patients prescribed on average >1 defined daily dose (DDD) during the one-year observational period had lower hospitalization risk after treatment initiation than those receiving lower doses (Appendix p6, pp14–15), aligning with trial evidence that higher psychostimulant doses may be more effective in reducing illicit use.^13^, ^14^, ^15^, ^16^, ^17^, ^18^, ^19^

Sensitivity analyses with shortened 8-week, 12-week and lengthened 12-month observation periods before and after treatment initiation confirmed findings of the primary analyses.

The sensitivity analyses in both sexes, different age groups, and different medication classes separately confirmed the finding that the rate of stimulant-related hospitalizations was not significantly higher following treatment initiation. It should be noted that even though the estimates for most subgroup analyses indicated a lower rate of stimulant-related hospitalizations after treatment initiation, the results were not all statistically significant, potentially because of the low number of hospitalizations in the subgroups and resulting wider CIs. Still, the consistent results of the primary and secondary analyses suggest that the results might be generalizable across sexes, age groups and different psychostimulant classes.

Several factors could contribute to the observed absence of increased stimulant-related hospitalizations among individuals with and without SUD history. First, prescription initiation involves comprehensive clinical assessment and monitoring, facilitating early identification and management of emerging complications. This medical oversight may prevent progression from minor problems to adverse events that require hospitalizations. Second, access to prescription stimulants under medical supervision may reduce use of illicit stimulant use and self-medication behaviors. For individuals with untreated ADHD symptoms, prescription treatment addresses underlying neurobiological deficits that might otherwise drive risk-taking behaviors. Third, prescription stimulants have pharmacokinetic properties that are favorable compared to illicit stimulants, including controlled-release mechanisms and standardized dosing that reduce overdose risk.

In individuals with SUD, treatment with psychostimulants may also function as substitution therapy, replacing hazardous illicit stimulant use patterns with medically supervised regimens. The dose–response relationship observed in our exploratory analyses, where higher defined daily doses showed greater hospitalization risk reduction, supports this substitution hypothesis and aligns with clinical trial evidence suggesting superior efficacy of higher stimulant doses in reducing illicit use. In individuals without a history of stimulant use disorder (SUD), our data reveal that prescription stimulant treatment was associated with a stable or slightly lower rate of stimulant-related hospitalizations compared to pre-treatment periods. This pattern appears less pronounced than in individuals with SUD history, but is noteworthy given background concerns about potential misuse in broader clinical populations. The relatively stable risk profile in those without SUD likely reflects both a lower baseline vulnerability to misuse and greater benefit from structured clinical monitoring and education that typically accompany the initiation of medical stimulant treatment. Epidemiological data indicate that while approximately 25% of adult prescription stimulant users report some form of misuse,^7^ the progression to clinically significant SUD remains limited in populations without prior SUD or misuse, and most incidents are mild in severity. In contrast, individuals with SUD history are at greater risk of harmful patterns of use. However, as our data and recent studies^13^, ^14^, ^15^, ^16^, ^17^, ^18^, ^19^ suggest, careful medical supervision and substitution therapy can substantially reduce severe complications. Thus, while both populations benefit from medical oversight, the magnitude of risk reduction is most evident among those with prior SUD, possibly reflecting higher benefits from stimulant substitution and structured clinical engagement.

Our findings of lower rates of stimulant-related hospitalizations after treatment initiation may appear contradictory to previous reports of relevant misuse rates among prescription stimulant users. However, these findings can be reconciled when considering the clinical spectrum of stimulant-related problems. Misuse encompasses a broad range of behaviors with varying clinical significance. Most documented misuse involves relatively mild behaviors such as occasional dose escalation or sharing medications—behaviors that infrequently result in hospitalization. In contrast, our outcome measure captured severe stimulant-related complications requiring hospital admission, representing the most serious clinical endpoint. The disconnect between high misuse rates and reduced hospitalizations suggests that while some misuse may occur, the overall clinical impact of prescription stimulant treatment on hospitalization rates may rather be protective than harmful.

The primary objective of our study was to assess the safety of psychostimulant treatment, rather than to evaluate efficacy. While it is possible that time-varying factors, such as clinical deterioration, could influence associations observed in our analyses, the key concern is whether prescribing stimulants precipitates excess risk of adverse outcomes. Our analyses indicate that the rate of stimulant-related hospitalizations is not higher in the 6-month period after treatment initiation, compared to the period before. Even if our observational associations are shaped in part by clinical context or supportive care, these results are reassuring for clinicians and patients.

Swedish and international data show a substantial increase in psychostimulant prescriptions over the last decades.^1^, ^2^, ^3^, ^4^, ^5^ The observed increases in ADHD medication prescribing may reflect not only improved recognition of ADHD by clinicians, but also increased public and parental awareness of ADHD, which may have prompted more individuals and families to seek diagnosis and treatment.^27^ While this greater awareness may benefit individuals with genuine impairment by facilitating access to appropriate treatment, concerns about potential overdiagnosis have also been raised.^28^ This underscores the importance of careful clinical assessment of ADHD diagnoses and consideration of potential adverse effects.

The presented findings should be interpreted with appropriate caution. Due to the observational nature of the data, our results cannot establish causal effects of treatment with psychostimulants or atomoxetine on SUD-related hospitalizations. Additionally, while our within-individual design eliminates confounding from time-invariant individual characteristics—offering control for selection bias and confounding by indication—it does not control for time-varying confounders. Potential time-varying factors include natural disease maturation, secular trends in healthcare provision, and changes in psychosocial circumstances around treatment initiation. These could have contributed to the observed associations and lower risks after treatment initiation. To reduce potential confounding by maturation or secular trends, outcome assessment was restricted to the first-year post-medication initiation and sensitivity analyses using time since index SUD diagnosis as covariate in the model, which however cannot control for trends driven by other factors. In addition, due to the required medication-free time period of at least two years, results might not be transferable to individuals with chronic long-term prescriptions of psychostimulants or atomoxetine. While long-term effects were beyond the study scope, previous research indicates no increased risk of substance misuse in longer follow-up periods.^26^

It should be noted that presented findings of our within-individual analyses apply to individuals with drug treatment who experienced a hospitalization before or after treatment initiation, but not to unexposed populations. Here, within-individual designs that explicitly control for time-varying covariates and target emulation trials provide methodological approaches that allow consideration of exposed and unexposed groups or periods respectively, thus allowing longer observational periods and estimation of treatment effect that might be more generalizable to the entire patient population and more robust against maturation effects. Still, the presented findings align with recent target trial emulation studies from Swedish register data evidencing reduced risk of adverse events, including self-harm, injury, traffic crashes and death, when individuals with ADHD received psychostimulants.^23^^,^^29^ The convergent findings across these methodological approaches strengthen confidence in treatment safety. Future research applying target trial emulation specifically to stimulant-related hospitalizations in SUD populations could confirm whether the reassuring safety profile generalizes to population-level estimates and longer observational periods.

We focused on adults (18–64 years), as they account for the majority of stimulant use and stimulant use disorders. This choice enhanced comparability between individuals with and without prior SUD and reduced heterogeneity related to developmental or age-related factors. The outcome was defined as stimulant-related hospitalizations, chosen for its clinical relevance to concerns about serious adverse effects, regardless of whether the stimulant was prescribed or illicit. We did not preselect individuals based on other psychiatric diagnoses, to allow examination of risks associated with stimulant prescribing in populations both with and without a history of SUD.

Although we did not expect substantial confounding from concomitant medications, we tested this by repeating the main analysis in individuals without overlapping antidepressant, sedative, or antipsychotic prescriptions around treatment initiation (±3 days) (Table 1), which confirmed the primary analysis (Appendix p16).

We also repeated the analyses using a narrower definition of stimulant-related hospitalizations—restricted to acute intoxication, harmful use, dependence, and withdrawal—and again observed results that corroborated the primary analysis (Appendix p17).

Taken together, our results may help to allay clinical concerns, as we found no indication of increased risk of stimulant-related hospitalizations following the initiation of psychostimulant treatment. Notably, this pattern was also observed among individuals with a history of SUD, in whom the risk appeared lower during treatment periods.

## Contributors

Patrick Bach, Härje Widing and Jonas Hällgren had full access to all the data in the study and take responsibility for the integrity of the data and the accuracy of the data analysis.

*Concept and design:* Patrick Bach, Jeanette Westman, Johan Franck, Mika Gissler.

*Acquisition, analysis, or interpretation of data:* Patrick Bach, Jeanette Westman, Johan Franck, Mika Gissler, Jonas Hällgren, Härje Widing.

*Drafting of the manuscript:* Patrick Bach.

*Critical review of the manuscript for important intellectual content:* Patrick Bach, Jeanette Westman, Johan Franck, Mika Gissler, Jonas Hällgren, Härje Widing.

*Statistical analysis:* Patrick Bach, Jonas Hällgren, Härje Widing.

*Administrative, technical, or material support:* Jeanette Westman, Johan Franck, Mika Gissler.

*Supervision:* Patrick Bach, Jeanette Westman, Johan Franck, Mika Gissler.

## Data sharing statement

No individual de-identified participant data will be shared. The data used in this study are derived from Swedish national registries, which are subject to strict confidentiality and data protection regulations. Access to these data is restricted by Swedish law and is granted only for specific research purposes following ethical approval and a data request process. No additional related documents, such as the study protocol or statistical analysis plan, will be made available beyond what is published in the article. Researchers interested in accessing similar data can apply to the relevant Swedish authorities, such as the National Board of Health and Welfare (Socialstyrelsen) and Statistics Sweden (SCB), following their established procedures. Access is granted on a case-by-case basis and requires an approved ethical review.

## Declaration of interests

Johan Franck and Jeanette Westman declare support for the current study as applicants for grants awarded by Forte (Dnr 2020-00467), Karolinska Institutet (KID, Dnr 2020-02653), and Region Stockholm (Dnr. FoUI-977024 and Dnr 2020-02653). Patrick Bach declares travel support for the current study by the German Center for Mental Health, Mannheim-Heidelberg-Ulm site. All other authors declare no competing interests. No other disclosures were reported.
