# Supplementary material for: Prescription psychostimulants or atomoxetine and the risk of stimulant-related hospital admissions in adults with and without stimulant use disorder: a Swedish population-based within-individual observational study
Source: Lancet Reg Health Eur. 2026 Mar 24;65:101658. doi: 10.1016/j.lanepe.2026.101658 (PMC13049997; doi:10.1016/j.lanepe.2026.101658)

**Supplementary appendix to**

*Prescription psychostimulants or atomoxetine and the risk of stimulant-related hospital admissions in adults with and without stimulant use disorder: a Swedish population-based within-individual observational study*

**Table 2.** List of the codes from the International Statistical Classification of Diseases and Related Health Problems 10th Revision (ICD-10) used to define the primary outcome of stimulant-related hospitalizations (F14.x and F15.x).

| **ICD-10 Code** | **Label** |
| --- | --- |
| **F14** | **Mental and Behavioral Disorders Due to Cocaine Use** |
| F14·0 | Mental and behavioral disorders due to use of cocaine: Acute intoxication |
| F14·1 | Mental and behavioral disorders due to use of cocaine: Harmful use |
| F14·2 | Mental and behavioral disorders due to use of cocaine: Dependence syndrome |
| F14·3 | Mental and behavioral disorders due to use of cocaine: Withdrawal state |
| F14·4 | Mental and behavioral disorders due to use of cocaine: Withdrawal state with delirium |
| F14·5 | Mental and behavioral disorders due to use of cocaine: Psychotic disorder |
| F14·6 | Mental and behavioral disorders due to use of cocaine: Amnesic syndrome |
| F14·7 | Mental and behavioral disorders due to use of cocaine: Residual and late-onset psychotic disorder |
| F14·8 | Mental and behavioral disorders due to use of cocaine: Other mental and behavioral disorders |
| F14·9 | Mental and behavioral disorders due to use of cocaine: Unspecified mental and behavioral disorder |
| **F15** | **Mental and Behavioral Disorders Due to Use of Other Stimulants, Including Caffeine** |
| F15·0 | Mental and behavioral disorders due to use of other stimulants, including caffeine: Acute intoxication |
| F15·1 | Mental and behavioral disorders due to use of other stimulants, including caffeine: Harmful use |
| F15·2 | Mental and behavioral disorders due to use of other stimulants, including caffeine: Dependence syndrome |
| F15·3 | Mental and behavioral disorders due to use of other stimulants, including caffeine: Withdrawal state |
| F15·4 | Mental and behavioral disorders due to use of other stimulants, including caffeine: Withdrawal state with delirium |
| F15·5 | Mental and behavioral disorders due to use of other stimulants, including caffeine: Psychotic disorder |
| F15·6 | Mental and behavioral disorders due to use of other stimulants, including caffeine: Amnesic syndrome |
| F15·7 | Mental and behavioral disorders due to use of other stimulants, including caffeine: Residual and late-onset psychotic disorder |
| F15·8 | Mental and behavioral disorders due to use of other stimulants, including caffeine: Other mental and behavioral disorders |
| F15·9 | Mental and behavioral disorders due to use of other stimulants, including caffeine: Unspecified mental and behavioral disorder |

## Table 3. Relative risk of stimulant use-related hospitalizations for individuals with (n=3 161) and without (n=129 505) history of SUD during the 8-week period after initiation of psychostimulant treatment compared to the 8-week period directly before treatment initiation. Period 2: the 8-week period before treatment initiation. Period 3: the 8-week period after treatment initiation. RR= Rate Ratio.

| **Period** | **Participants with at least one hospitalization** | **Number of hospitalizations** | **RR (95% CI)** |
| --- | --- | --- | --- |
| **Individuals with a history of SUD (n=3 161)** | | |  |
| 2 | 41 | 53 | 1 (reference) |
| 3 | 42 | 46 | 0·87 (0·58-1·29) |
| **Individuals without a history of SUD (n=129 505)** | | |  |
| 2 | 66 | 69 | 1 (reference) |
| 3 | 56 | 56 | 0·81 (0·57-1·15) |

## Table 4. Relative risk of stimulant use-related hospitalizations for individuals with (n=3 161) and without (n=129 505) history of SUD during the 12-week period after initiation of psychostimulant treatment compared to the 12-week period directly before treatment initiation. Period 2: the 12-week period before treatment initiation. Period 3: the 12-week period after treatment initiation. RR= Rate Ratio.

| **Period** | **Participants with at least one hospitalization** | **Number of hospitalizations** | **RR (95% CI)** |
| --- | --- | --- | --- |
| **Individuals with a history of SUD (n=3 161)** | | |  |
| 2 | 59 | 81 | 1 (reference) |
| 3 | 55 | 61 | 0·75 (0·54-1·05) |
| **Individuals without a history of SUD (n=129 505)** | | |  |
| 2 | 97 | 110 | 1 (reference) |
| 3 | 91 | 94 | 0·85 (0·65-1·13) |

## Table 5. Relative risk of stimulant use-related hospitalizations for individuals with (n=3 161) and without (n=129 505) history of SUD during the 12-month period after initiation of psychostimulant treatment compared to the 12-month period directly before treatment initiation. Period 2: the 12-month period before treatment initiation. Period 3: the 12-month period after treatment initiation. RR= Rate Ratio.

| **Period** | **Participants with at least one hospitalization** | **Number of hospitalizations** | **RR (95% CI)** |
| --- | --- | --- | --- |
| **Individuals with a history of SUD (n=3 161)** | | |  |
| 2 | 203 | 345 | 1 (reference) |
| 3 | 153 | 234 | 0·68 (0·57-0·80) |
| **Individuals without a history of SUD (n=129 505)** | | |  |
| 2 | 356 | 441 | 1 (reference) |
| 3 | 328 | 402 | 0·91 (0·79-1·04) |

## Table 6. Definition of defined daily doses (DDDs) for psychostimulants and atomoxetine together with units, routes of administration, and additional notes based on data from the WHO Collaborating Centre for Drug Statistics Methodology.

| ATC code | Name | DDD | Unit | Route of Administration |
| --- | --- | --- | --- | --- |
| N06BA01 | Amphetamine | 15 | mg | Oral |
| N06BA02 | Dexamphetamine | 15 | mg | Oral |
| N06BA03 | Methamphetamine | 15 | mg | Oral |
| N06BA04 | Methylphenidate | 30 | mg | Oral |
| N06BA07 | Modafinil | 0·3 | g | Oral |
| N06BA09 | Atomoxetine | 80 | mg | Oral |
| N06BA11 | Dexmethylphenidate | 15 | mg | Oral |
| N06BA12 | Lis-dexamphetamine | 30 | mg | Oral |
| N06BA13 | Armodafinil | 0·15 | g | Oral |

## ATC code: Anatomical Therapeutic Chemical classification code, Name: International nonproprietary name, DDD: Defined Daily Dose

## Table 7. Relative risk of stimulant use-related hospitalizations for individuals with (n=3 161) history of SUD in period 1, period 3, and period 4, compared to period 2 (reference) adjusted for the time since index diagnosis of a stimulant use disorder in the study period. Period 1: the 6-month period starting 1 calendar year before treatment initiation. Period 2: the 6-month period before treatment initiation. Period 3: the 6-month period after treatment initiation. Period 4: the 6-month period starting 6 months after treatment initiation. RR= Rate Ratio.

| **Period** | **Participants with at least one hospitalization** | **Number of hospitalizations** | **RR (95% CI)** |
| --- | --- | --- | --- |
| **Individuals with a history of SUD (n=3 161)** | | |  |
| 1 | 116 | 181 | 0·99 (0·73-1·35) |
| 2 | 119 | 164 | 1 (reference) |
| 3 | 97 | 129 | 0·71 (0·52-0·96) |
| 4 | 82 | 105 | 0·51 (0·32-0·82) |

## Figure 3. Relative risk of stimulant use-related hospitalizations for different medication subgroups in individuals with a history of stimulant use disorder (n=3 161) in period 1, period 3, and period 4, compared to period 2 (reference). Period 1: the 6-month period starting 1 calendar year before treatment initiation. Period 2: the 6-month period before treatment initiation. Period 3: the 6-month period after treatment initiation. Period 4: the 6-month period starting 6 months after treatment initiation. RR = Rate Ratio.


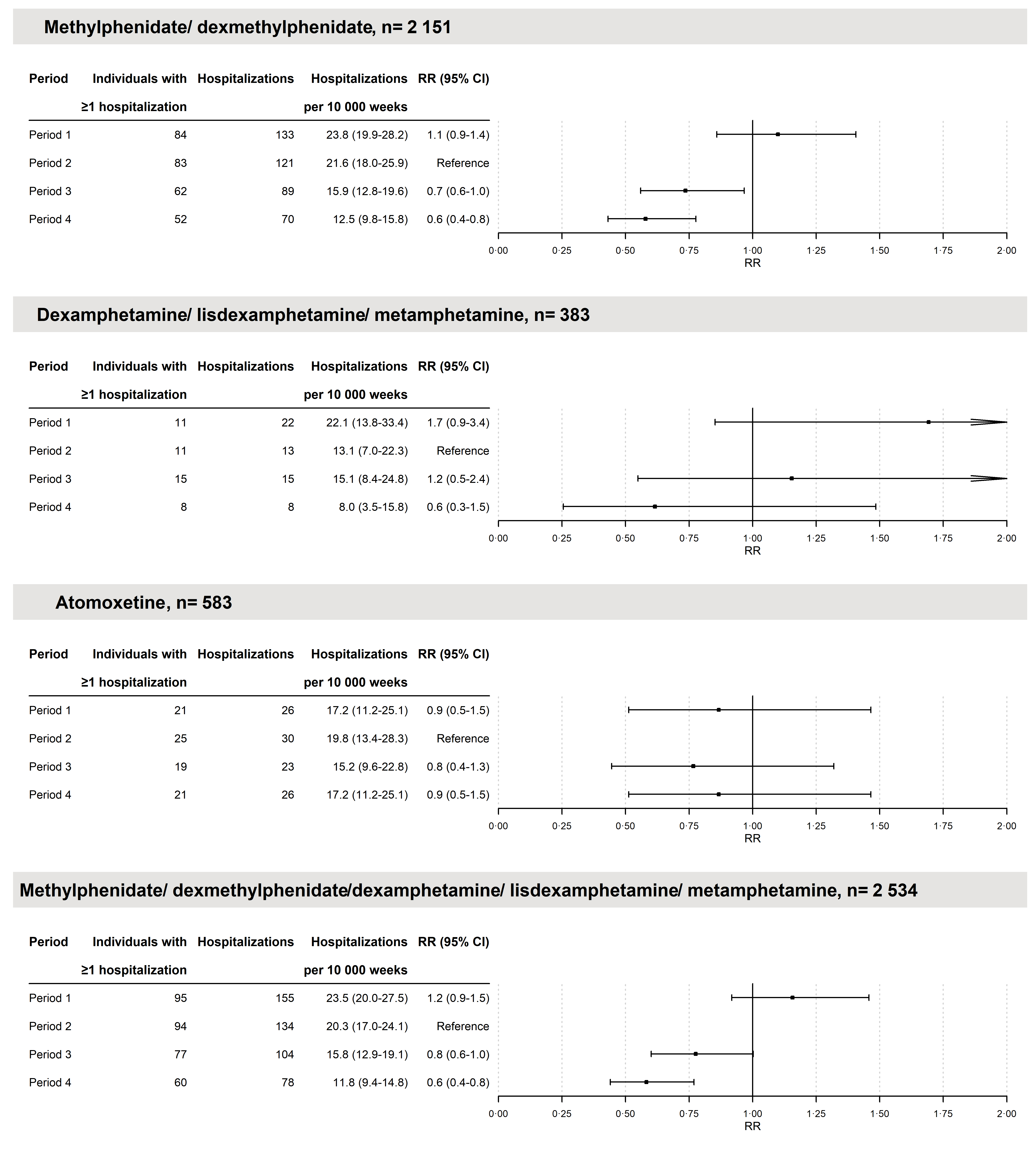


## Figure 4. Relative risk of stimulant use-related hospitalizations for different medication subgroups in individuals without history of stimulant use disorder (n=129 505) in period 1, period 3, and period 4, compared to period 2 (reference). Period 1: the 6-month period starting 1 calendar year before treatment initiation. Period 2: the 6-month period before treatment initiation. Period 3: the 6-month period after treatment initiation. Period 4: the 6-month period starting 6 months after treatment initiation. RR = Rate Ratio.

**
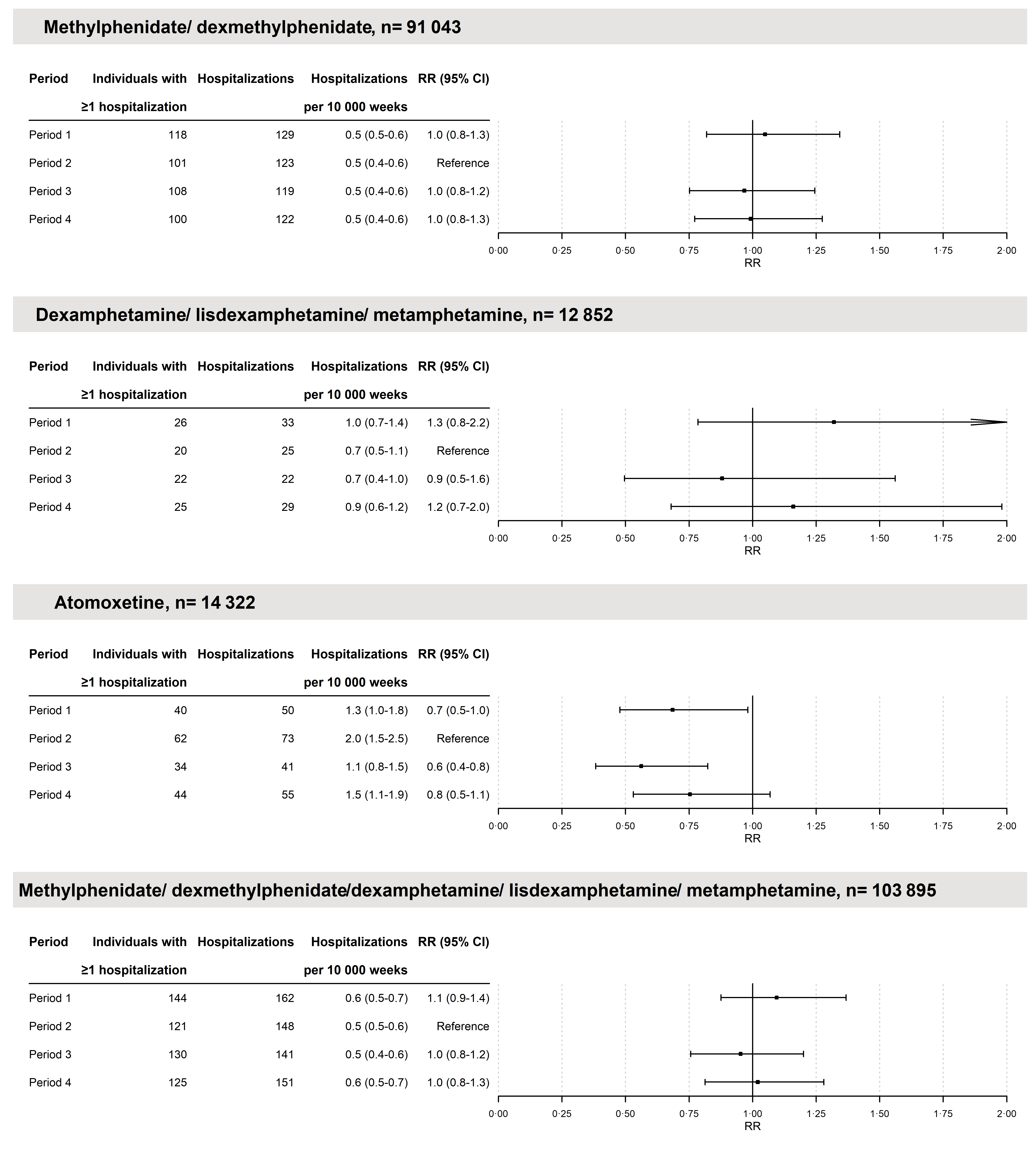
**

## Figure 5. Relative risk of stimulant use-related hospitalizations among females (n=2 088) and males (n=1 073) with history of a stimulant use disorder during different periods before (period 1) and after initiation (period 3 and 4) of treatment (reference: period 2). RR = Rate Ratio.


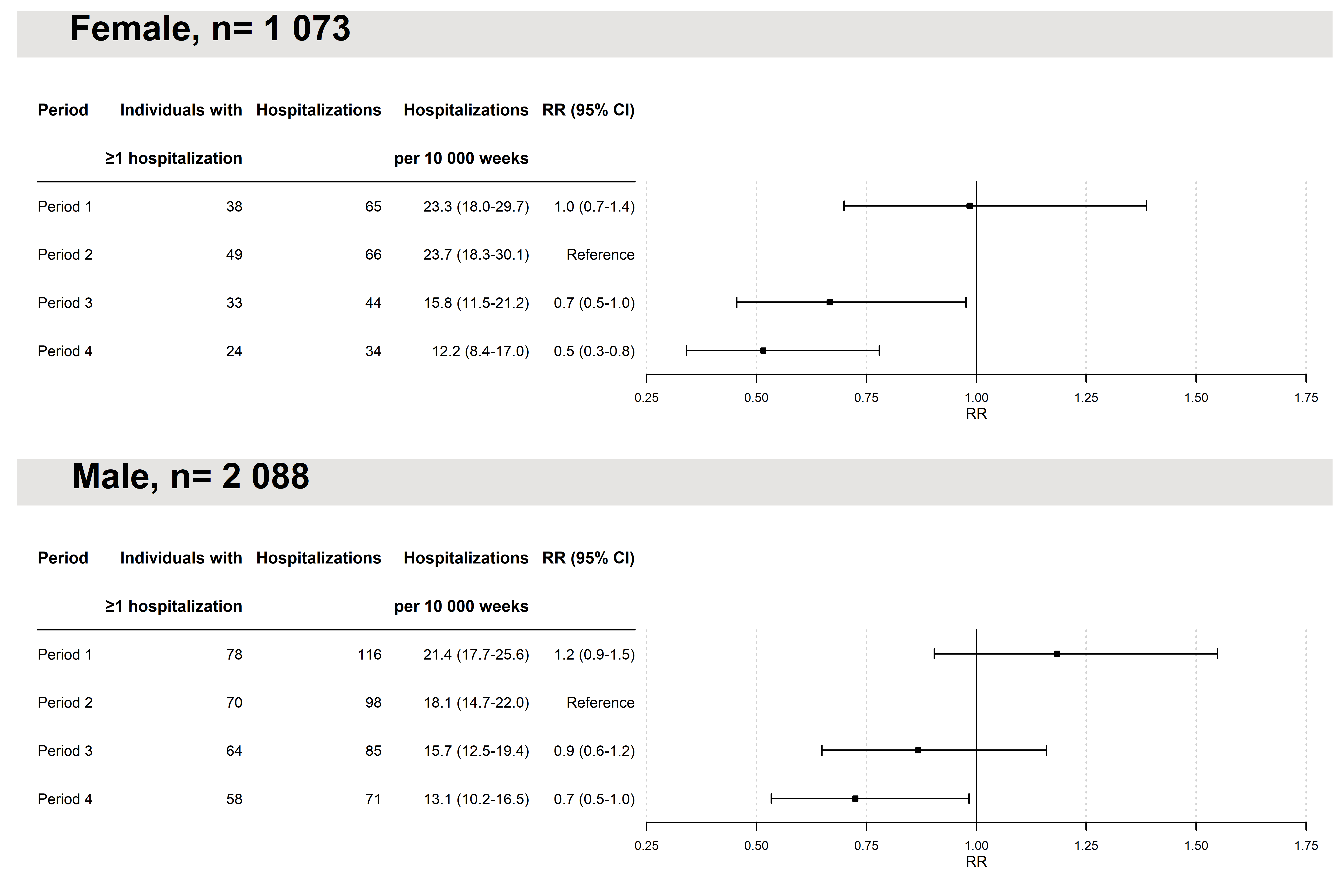


## Figure 6. Relative risk of stimulant use-related hospitalizations among females (n=64 717) and males (n=64 788) without a history of a stimulant use disorder during different periods before (period 1) and after initiation (period 3 and 4) of treatment (reference: period 2). RR = Rate Ratio.


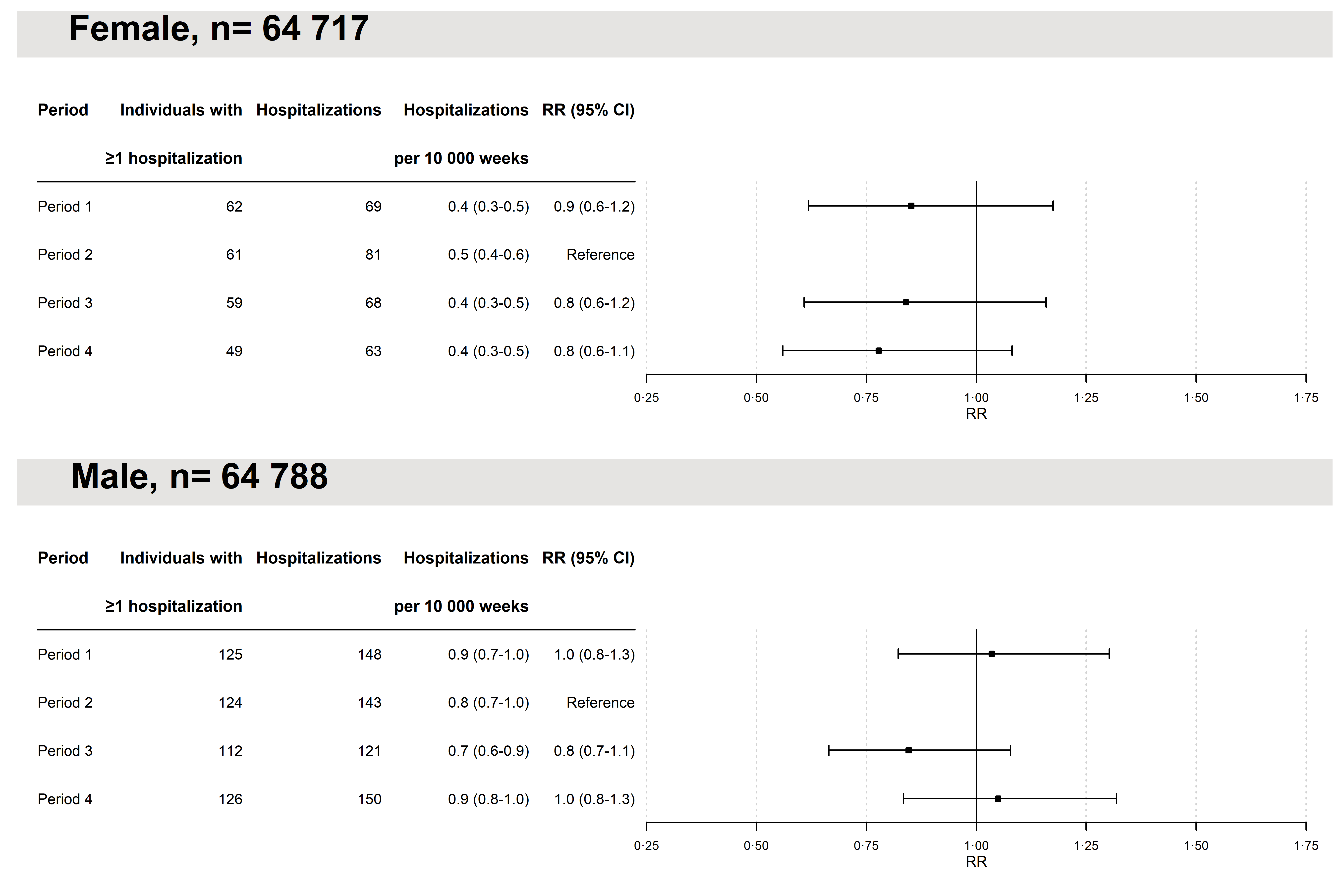


## Figure 7. Depiction of the relative risk of stimulant use-related hospitalizations among individuals with history of stimulant use disorder aged 18 to 34 years with (N=1 550), 35 to 49 years (N= 1 231) and 50 to 64 years (N=380) during the periods before (period 1) and after initiation (period 3 and 4) of treatment (reference: period 2). RR= Rate Ratio.


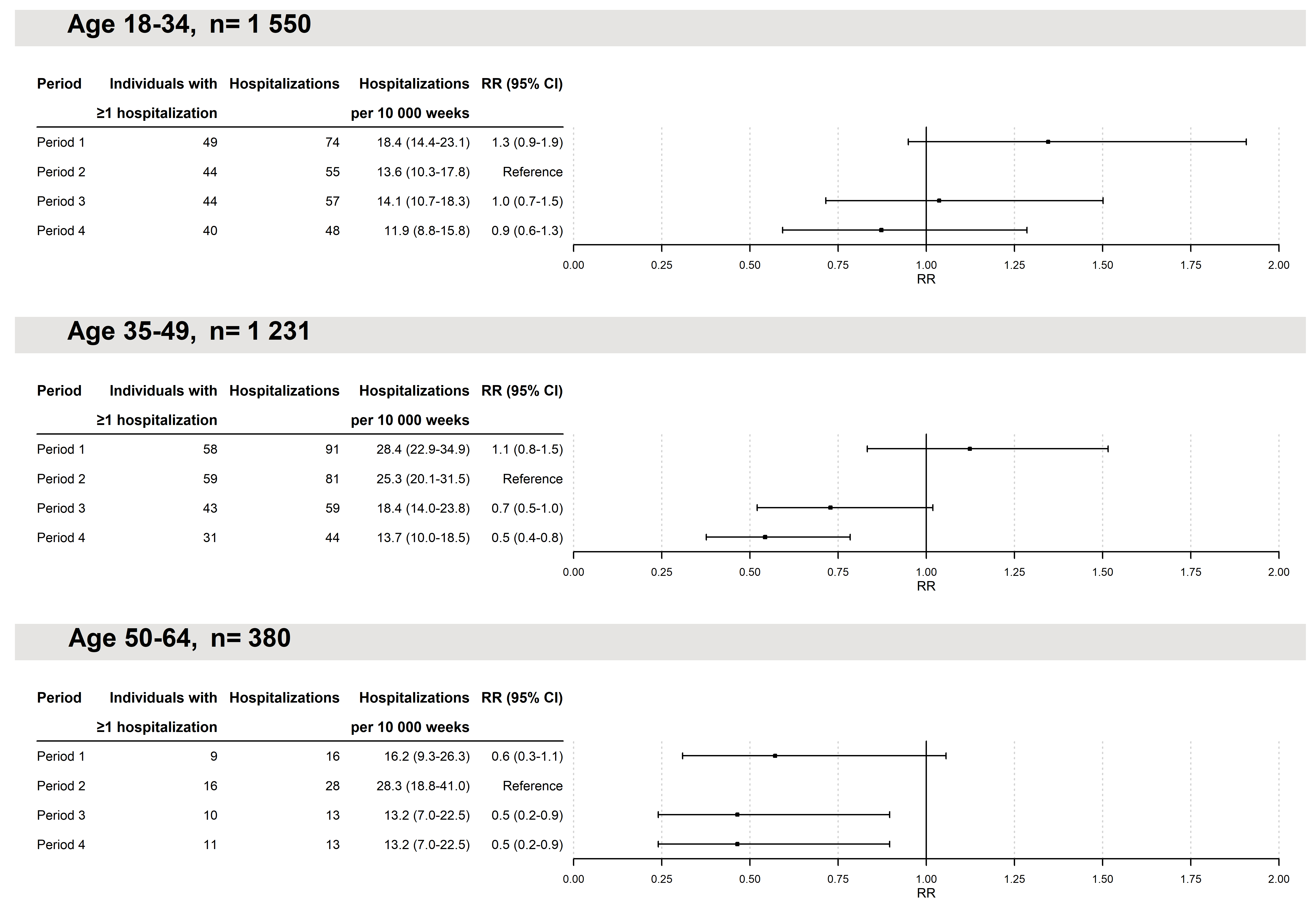


## Figure 8. Depiction of the relative risk of stimulant use-related hospitalizations among individuals with without history of stimulant use disorder aged 18 to 34 years with (N=74 792), 35 to 49 years (N=40 788) and 50 to 64 years (N=13 675) during the periods before (period 1) and after initiation (period 3 and 4) of treatment (reference: period 2). RR= Rate Ratio


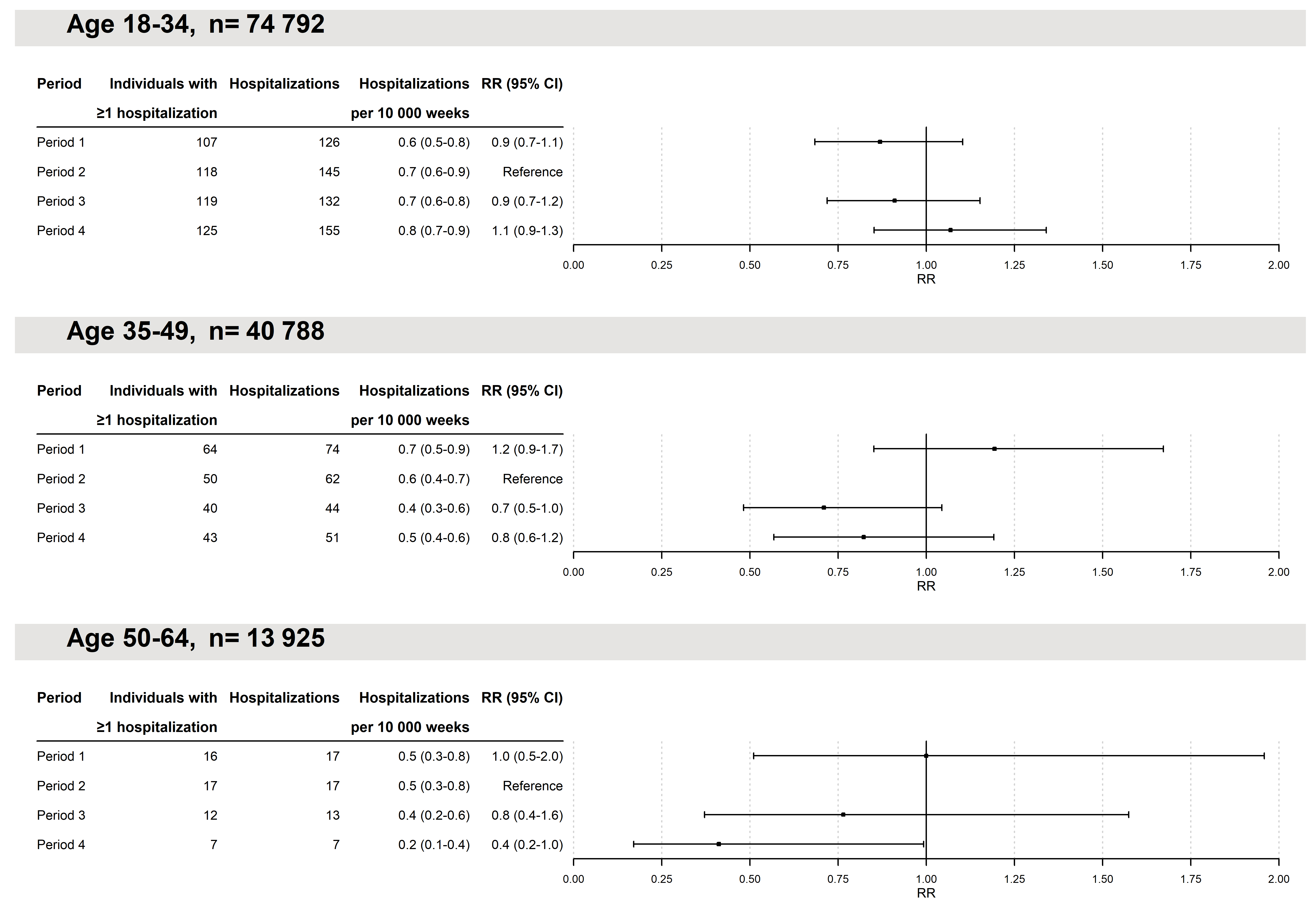


## Figure 9. Relative risk of stimulant use-related hospitalizations among individuals with history of stimulant use disorder, who received on average > 1 defined daily dose (DDD) during the one-year observational period and those reviving < 1 DDD dose during different periods before (period 1) and after initiation (period 3 and 4) of treatment (reference: period 2). RR = Rate Ratio.


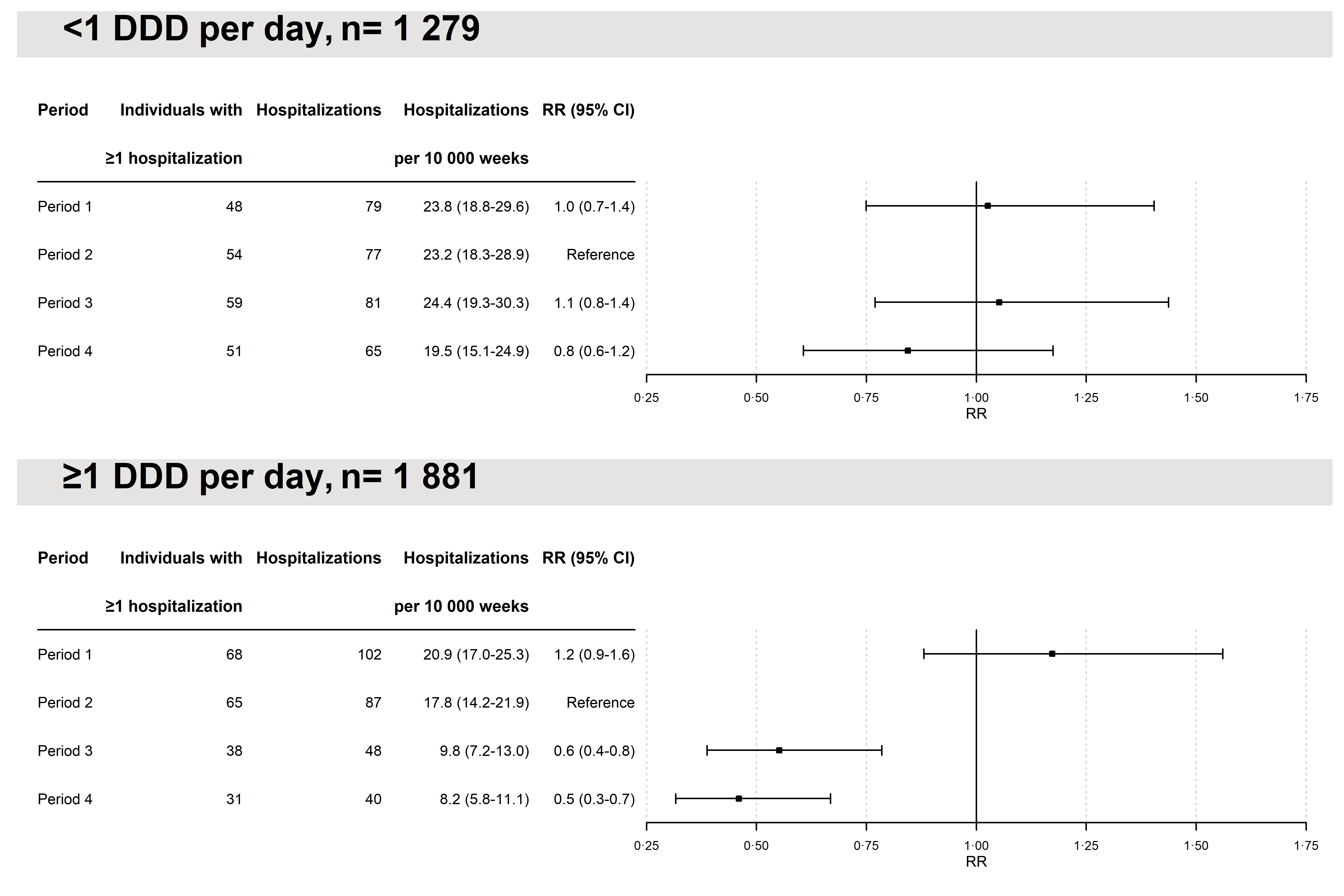


## Figure 10. Relative risk of stimulant use-related hospitalizations among individuals without history of stimulant use disorder, who received on average > 1 defined daily dose (DDD) during the one-year observational period and those reviving < 1 DDD dose during different periods before (period 1) and after initiation (period 3 and 4) of treatment (reference: period 2). RR = Rate Ratio.


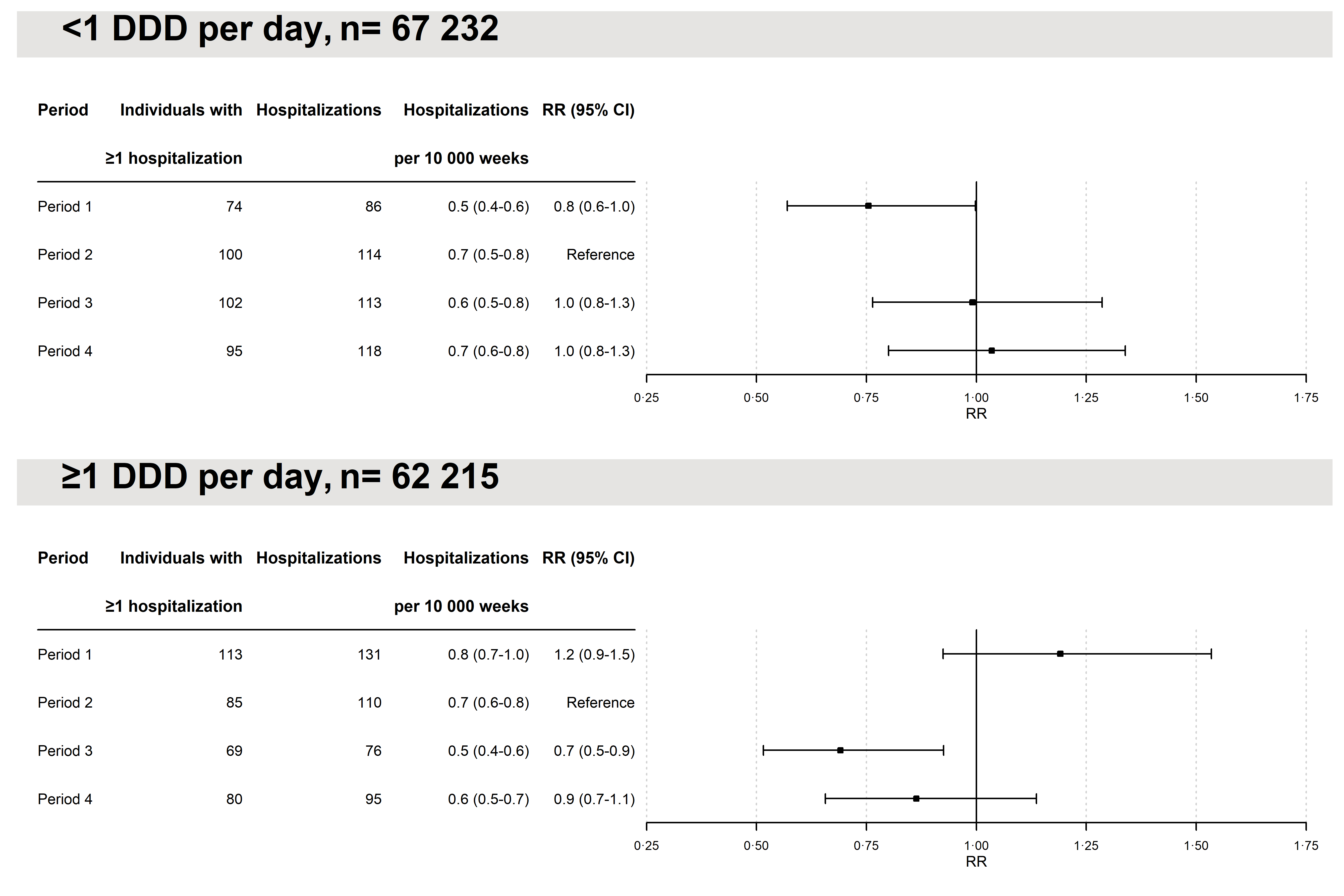


## Figure 11. Relative risk of stimulant use-related hospitalizations among individuals who did not receive antipsychotics, antidepressants, or sedatives +/- 3 days before or after initiation of treatment during different periods before (period 1) and after initiation (period 3 and 4) of treatment (reference: period 2). Results are shown for individuals with (n=2 244) and without (n=102 114) a history of stimulant use disorder. RR = Rate Ratio.


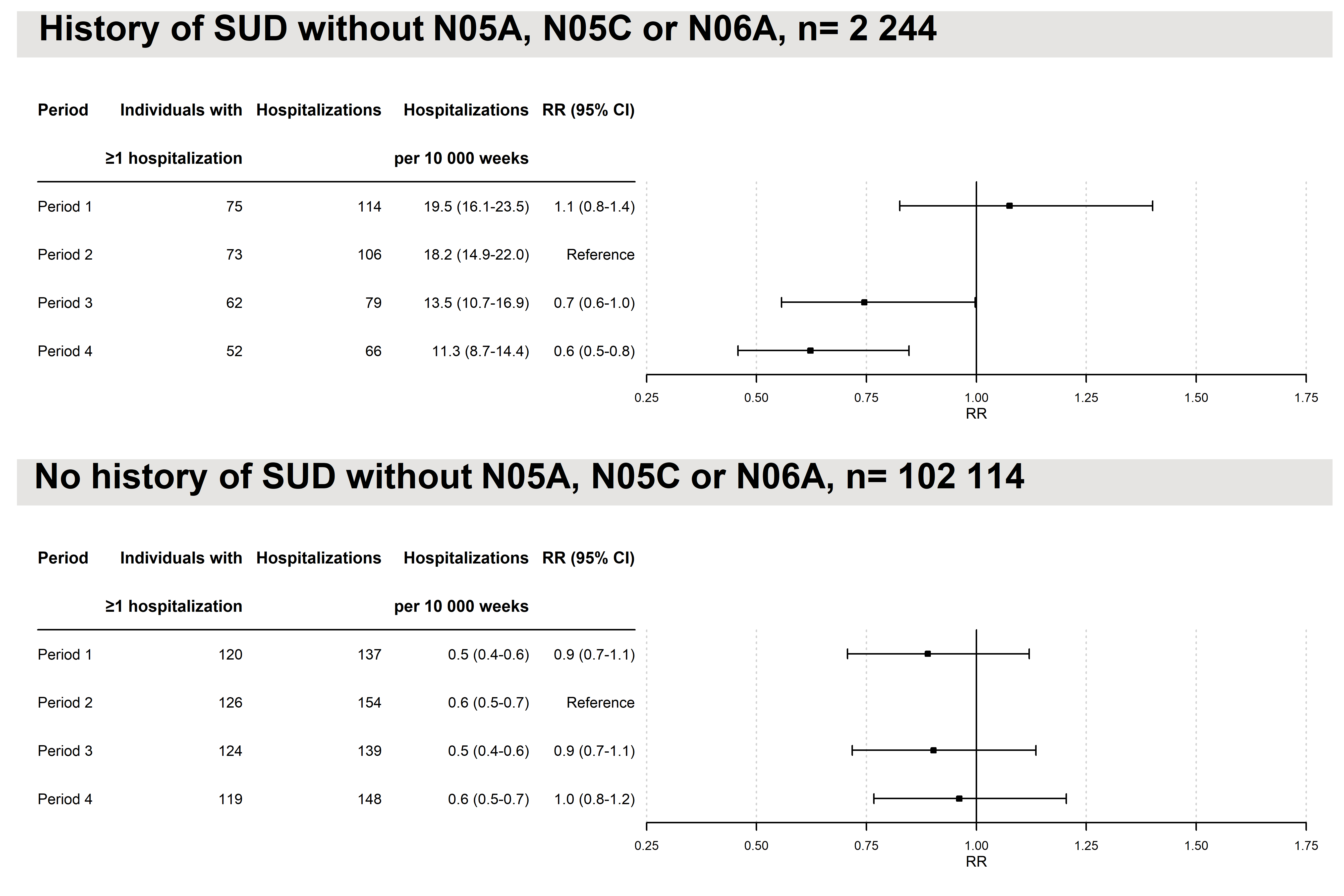


## Figure 12. Relative risk of hospital admissions due to stimulant intoxication (ICD-10 codes: F14.0 or F15.0), stimulant abuse (ICD-10 codes: F14.1 or F15.1), stimulant dependence (ICD-10 codes: F14.2 or F15.2), stimulant withdrawal syndrome without delirium (ICD-10 codes: F14.3 or F15.3), and stimulant withdrawal with delirium (ICD-10 codes: F14.4 or F15.4) during different periods before (period 1) and after initiation (period 3 and 4) of psychostimulant treatment (reference: period 2). Results are shown individuals with (n=3 161) and without (n=129 505) history of a stimulant use disorder. RR = Rate Ratio.


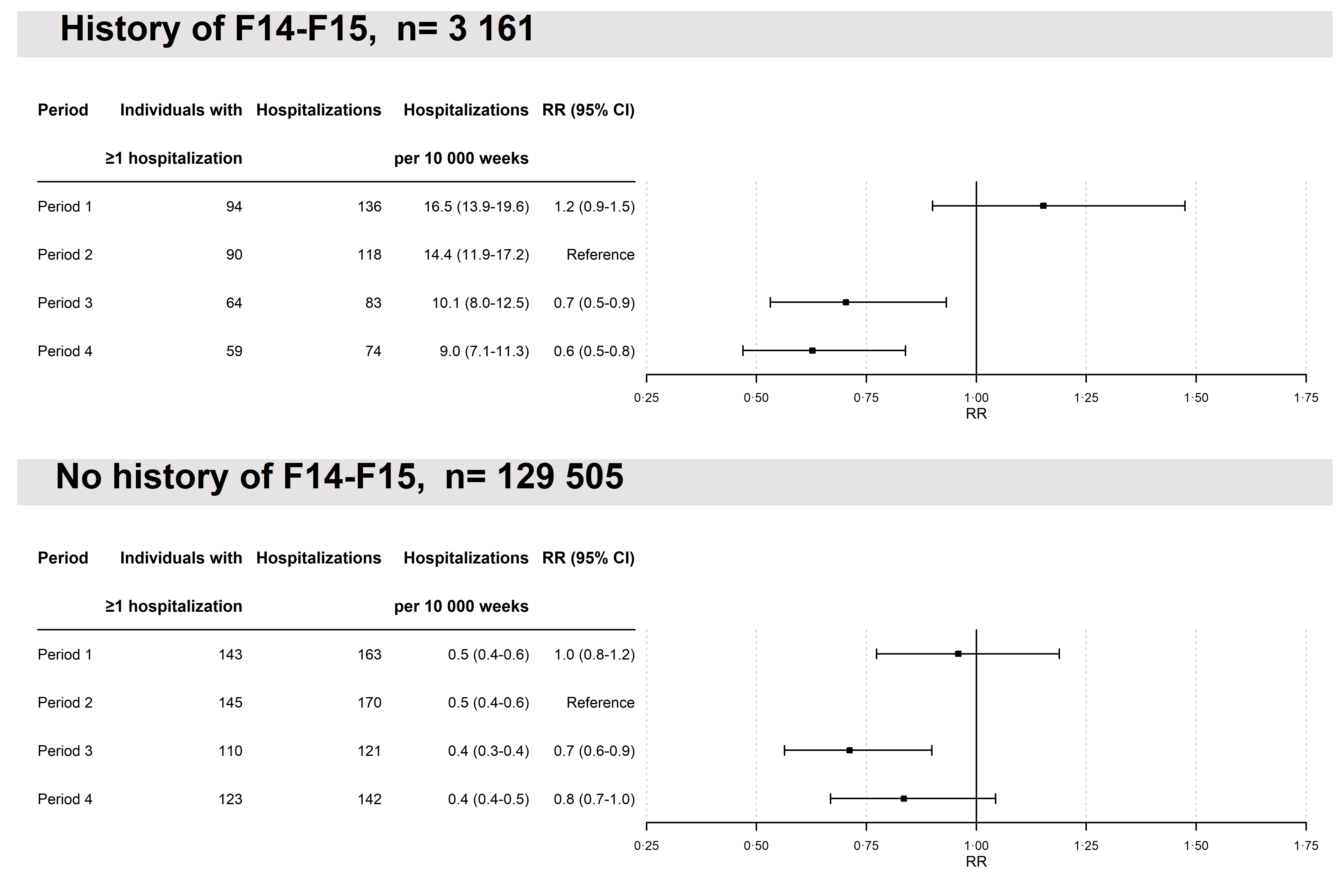

Supplement: Appendix [file mmc1.docx]
